# Mixed Infectious–Immune Pneumonitis Associated with PD-L1 Blockade: A Case of Durvalumab-Induced Lung Injury Complicated by Human Metapneumovirus Infection

**DOI:** 10.3390/jcm15010251

**Published:** 2025-12-29

**Authors:** Luca Pipitò, Chiara Vincenza Mazzola, Ilenia Giacchino, Riccardo De Rosa, Carola Maria Gagliardo, Alessio Giuseppe Lipari, Paola Monte, Federica Furia, Erika Mannino, Rosaria Pecoraro, Nicola Scichilone, Antonio Cascio

**Affiliations:** 1 Department of Health Promotion, Mother and Child Care, Internal Medicine and Medical Specialties, University of Palermo, 90127 Palermo, Italy; chiaravincenza.mazzola@community.unipa.it (C.V.M.); ilenia.giacchino@unipa.it (I.G.); riccardo.derosa@community.unipa.it (R.D.R.); carolamaria.gagliardo@you.unipa.it (C.M.G.); alessiogiuseppe.lipari@you.unipa.it (A.G.L.); paola.monte@you.unipa.it (P.M.); federica.furia@you.unipa.it (F.F.); manninoerika@gmail.com (E.M.);; 2 Infectious and Tropical Disease Unit, AOU Policlinico “P. Giaccone”, 90127 Palermo, Italy; 3 Microbiology and Virology Unit, AOU Policlinico “P. Giaccone”, 90127 Palermo, Italy; 4 U.O.C di Medicina Interna con Stroke Care, AOU Policlinico “P. Giaccone”, 90127 Palermo, Italy; rosaria.pecoraro@policlinico.pa.it

**Keywords:** durvalumab, PD-L1 inhibitor, immune checkpoint inhibitor pneumonitis, Human metapneumovirus (HMPV), immune-related adverse events (irAEs), mixed infectious–immune pneumonitis

## Abstract

**Background:** Durvalumab, a PD-L1 inhibitor used as consolidation therapy after chemoradiation in unresectable stage III non–small cell lung cancer (NSCLC), can induce immune-related adverse events, among which immune-mediated pneumonitis represents one of the most severe. Differentiating checkpoint inhibitor pneumonitis (CIP) from infectious pneumonia is challenging due to overlapping clinical and radiologic findings. **Case presentation:** We describe a 67-year-old woman with stage III lung adenocarcinoma treated with chemotherapy, radiotherapy, and durvalumab, who presented with progressive dyspnea and extensive bilateral ground-glass opacities on CT imaging. Laboratory tests revealed leukopenia and elevated inflammatory markers. Despite broad-spectrum antibiotic and antiviral therapy, her condition worsened, requiring high-flow nasal cannula oxygen therapy. Multiplex molecular testing on sputum identified human metapneumovirus (HMPV), while blood cultures and urinary antigens for *Streptococcus pneumoniae* and *Legionella pneumophila* were negative. A pulmonology consultation raised suspicion for severe durvalumab-induced pneumonitis exacerbated by viral infection. High-dose methylprednisolone (2 mg/kg/day) followed by a four-week taper led to gradual clinical and radiologic resolution. Durvalumab was permanently discontinued. **Discussion:** To our knowledge, this is the first reported case of HMPV-associated pneumonitis in a patient receiving durvalumab. This case highlights the potential synergistic interplay between viral infection and immune checkpoint blockade, resulting in severe lung injury. Comprehensive microbiologic evaluation, including molecular diagnostics, is essential to guide therapy and distinguish infectious from immune-mediated causes. **Conclusions:** Early recognition of mixed infectious and immune-mediated pneumonitis, and timely corticosteroid therapy are critical to achieving favorable outcomes and preventing irreversible pulmonary damage.

## 1. Introduction

Immune checkpoint inhibitors (ICIs) targeting the programmed cell death 1 (PD-1)/programmed death-ligand 1 (PD-L1) axis have revolutionized the management of advanced and locally advanced non–small cell lung cancer (NSCLC), significantly improving overall survival and long-term disease control [1,2].

Durvalumab, a fully human monoclonal antibody targeting PD-L1, prevents its interaction with PD-1 and CD-80, thereby enhancing antitumor immune responses by sustaining T-cell activation and proliferation [3]. Despite its proven efficacy as consolidation therapy after chemoradiation in unresectable stage III NSCLC, durvalumab, like other ICIs, can induce a range of immune-related adverse events (irAEs) due to nonspecific immune activation against healthy tissues [2,4,5,6,7].

Among these, immune-mediated pneumonitis is one of the most serious and potentially life-threatening toxicities [2,4,5,6,7]. Clinical manifestations are often nonspecific, including cough, dyspnea, and low-grade fever, while radiologic findings may overlap with infectious pneumonia, tumor progression, or radiation-induced lung injury [2]. This overlap complicates the differential diagnosis and underscores the need for a comprehensive assessment integrating clinical, radiologic, and microbiologic data [2].

Here, we present a case of severe community-acquired pneumonia (CAP) with a mixed infectious and immune-mediated etiopathogenesis, in which human metapneumovirus (HMPV) infection coexisted with durvalumab-induced pneumonitis. To our knowledge, this represents the first reported case of HMPV-associated pneumonitis in a patient receiving durvalumab therapy.

## 2. Case Presentation

In January 2025, a 67-year-old woman, a former smoker with an approximately 40 pack-year smoking history who had quit two years earlier, presented to the emergency department with progressive dyspnea, chest pain, and easy fatigability over several days, without fever.

In December 2023, she had been diagnosed with lung adenocarcinoma, treated with chemotherapy and radiotherapy, and was under follow-up at our Oncology Department. For the previous three months, she had been receiving immunotherapy with durvalumab, administered every 14 days (the last infusion two weeks before presentation). The patient received a total of six cycles of durvalumab. Her medical history was also remarkable for paroxysmal atrial fibrillation treated with apixaban, and polymyalgia rheumatica, previously managed with weekly methotrexate. The latter was self-discontinued by the patient due to excessive hair loss. Her Eastern Cooperative Oncology Group performance status was 2. She was ambulatory and capable of all self-care but unable to carry out any work-related activities.

**Figure 1:**
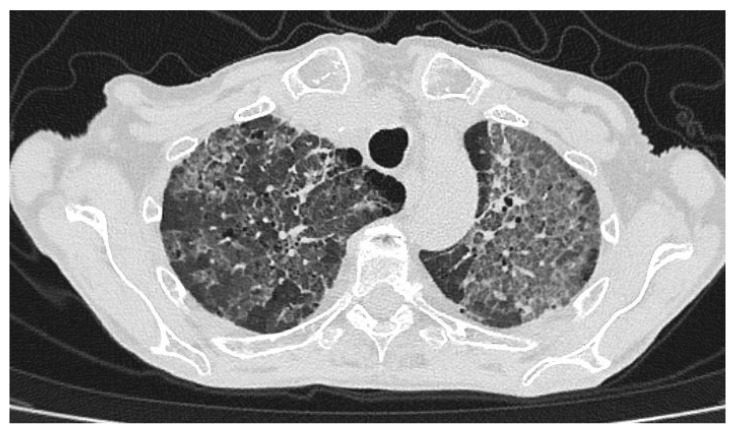
Chest CT scan shows scattered areas of ground-glass opacity predominantly involving both upper lobes, the middle lobe, the lingula, and the left lower lobe.

At presentation, the patient’s body temperature was 37.5 °C, blood pressure 90/60 mmHg, pulse rate 84 bpm, oxygen saturation (SpO_2_) 91% on 3 L/min O_2_ via simple face mask, and respiratory rate 28 breaths/min. Chest computed tomography (CT) demonstrated multiple, extensive ground-glass opacities predominantly involving both upper lobes, the middle lobe, the lingula, and the left lower lobe (Figure 1). Laboratory results revealed white blood cell count (WBC) 4360/mm^3^ (reference range 4000–11,000/mm^3^) with 86% neutrophils (reference range 40–74%), and C-reactive protein (CRP) 35 mg/L (reference < 5 mg/L). Arterial blood gas analysis (on room air): pH 7.33, pO_2_ 50 mmHg, pCO_2_ 33 mmHg, HCO_3_^−^ 17.4 mmol/L, lactate 1.3 mmol/L, SaO_2_ 86%. Other chemical examinations were unremarkable. Serology for human immunodeficiency virus was negative.

The patient was admitted to the infectious diseases unit with a working diagnosis of CAP.

Empirical antibiotic and antiviral therapy was initiated with ceftobiprole 500 mg three times daily, doxycycline 100 mg twice daily, and oseltamivir 75 mg twice daily. Despite therapy, no significant clinical improvement was observed, and within a few days her respiratory status deteriorated, requiring escalation of oxygen therapy first to a Venturi mask (FiO_2_ 60%), and subsequently to high-flow nasal cannula (HFNC) at 40 L/min on the fifth day of hospitalization. Concurrently, inflammatory markers worsened, with CRP peaking at 96.1 mg/L and procalcitonin 0.293 µg/L (reference < 0.05 µg/L). CT angiography was negative for pulmonary embolism.

On the fourth hospital day, an extensive microbiological work-up was performed, including a multiplex molecular panel (BIOFIRE^®^ FILMARRAY^®^ Pneumonia Panel Plus, bioMérieux, Salt Lake City, UT, USA), which detects 27 bacterial and viral respiratory pathogens as well as seven antibiotic resistance genes on nasopharyngeal swab and sputum [8]. Additionally, urinary antigens for *Streptococcus pneumoniae* and *Legionella pneumophila*, and three sets of blood cultures were performed.

The molecular panel identified HMPV in the sputum, while it was negative on the nasopharyngeal swab. Sputum and blood cultures remained negative, and urinary antigens were also negative.

After a pulmonology consultation, the patient was suspected to have severe durvalumab-induced immune-mediated pneumonitis, potentially exacerbated by HMPV infection, based on recent durvalumab exposure and typical radiologic findings.

Methylprednisolone 40 mg twice daily (2 mg/kg per day) was administered for 10 days, followed by a 14-day tapering regimen, with subsequent clinical improvement. Antibiotic therapy was discontinued after 7 days. During hospitalization, the patient developed leukopenia, attributed to durvalumab, with a nadir WBC of 1050/mm^3^ on day 5 (neutrophils 70.5%). Progressive normalization was later observed, reaching 6520/mm^3^ at discharge.

Gradually, the patient was successfully weaned off HFNC, and on the 15th day, continued oxygen support via nasal cannula; finally, oxygen therapy was discontinued. After 18 days of hospitalization, the patient was discharged in good clinical condition and on room air.

**Figure 2:**
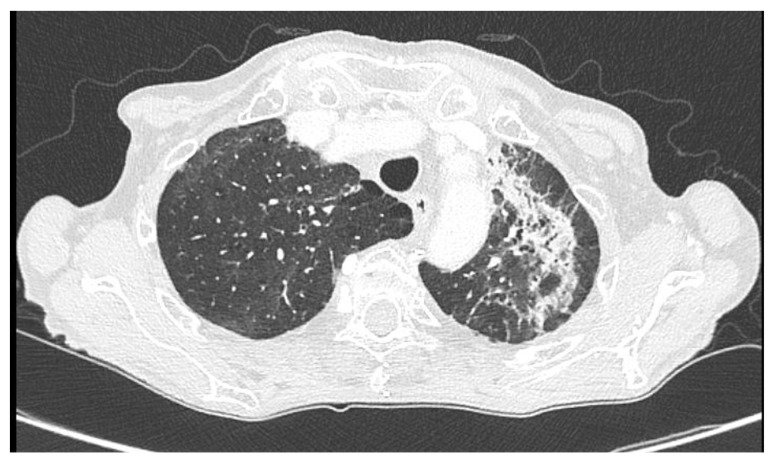
Follow-up contrast-enhanced chest CT performed 4 months after discharge, showing a marked reduction in areas of increased parenchymal density compared with the prior scan.

Four months after discharge, the patient underwent a contrast-enhanced whole-body CT scan for oncologic follow-up, which showed a marked reduction in areas of increased parenchymal density compared with the prior CT (Figure 2).

At the follow-up oncology visit, the oncologist, agreeing with the diagnosis of severe durvalumab-induced immune-mediated pneumonitis, decided not to reintroduce the drug.

The patient did not resume durvalumab therapy thereafter, and no other ICIs were administered during the 10 months following discharge.

## 3. Discussion

PD-L1 inhibitor–associated severe pneumonitis is a well-documented immune-related adverse event. Patients diagnosed with irAEs in the emergency department generally present with higher-grade toxicities, and approximately 3.5% of patients with severe irAEs require hospitalization and corticosteroid treatment [1,2].

The condition may manifest at any time during therapy, although onset typically occurs within 6–12 weeks after initiation [2]. In our patient, symptoms developed approximately three months after starting durvalumab, consistent with the expected temporal pattern. Prior thoracic chemoradiation likely contributed to increased susceptibility to pulmonary toxicity, as reported in several studies describing synergistic lung injury mechanisms between radiation-induced fibrosis and immune activation [1]. Furthermore, recent retrospective analyses have identified male sex and pre-existing autoimmune disorders as potential risk factors for severe pneumonitis during durvalumab therapy [9]. Our patient’s history of polymyalgia rheumatica, albeit inactive, may have predisposed her to dysregulated immune activation, further contributing to the development of pneumonitis.

The differential diagnosis between infectious pneumonia and CIP remains challenging in patients receiving immune checkpoint inhibitors, especially when presenting with fever and elevated inflammatory markers. The integration of molecular diagnostics provides significant clinical value in this context. This technology enables rapid detection of bacterial and viral pathogens even after the initiation of empirical antimicrobial therapy, improving diagnostic accuracy and enabling appropriate therapeutic adjustments. In our case, molecular testing was decisive in revealing a mixed infectious–immune etiology, guiding the discontinuation of antibiotics and the initiation of corticosteroid therapy.

The clinical presentation of CIP is often nonspecific, including dyspnea, cough, and fatigue, and may closely resemble infectious pneumonia or tumor progression [2]. In this case, the patient presented with progressive dyspnea, mild fever, and extensive ground-glass opacities on CT imaging, initially suggesting CAP. The absence of bacterial pathogens in sputum and blood cultures, along with negative urinary antigen tests and a poor response to broad-spectrum antibiotics and antiviral therapy, raised suspicion of immune-mediated pneumonitis. The identification of HMPV through multiplex PCR testing, however, introduced an additional layer of complexity.

HMPV is increasingly recognized as a cause of lower respiratory tract infection in adults, particularly in immunocompromised and oncologic patients [10,11,12]. The detection of HMPV exclusively in the sputum sample, but not in the nasopharyngeal swab, supports its localization in the lower respiratory tract and strengthens its pathogenic role.

Currently, no specific antiviral therapy is approved for HMPV. Management is primarily supportive. Agents such as ribavirin or immunoglobulin preparations have been used in selected or severely immunocompromised patients, but evidence for their efficacy remains limited [13].

Although HMPV can cause severe pneumonia on its own, its presence in this case may have acted as a trigger or amplifier of the immune-mediated pulmonary inflammation initiated by durvalumab.

This co-detection underscores the potential interaction between viral infection and immune checkpoint blockade, in which viral activation may amplify pulmonary inflammation or trigger immune-mediated toxicity. To the best of our knowledge, our case is the first reported in the literature of CIP in a patient receiving durvalumab complicated by HMPV infection. Previous reports have described the coexistence of viral infections and ICI-induced pneumonitis. Several cases of influenza and SARS-CoV-2 infections have been reported during ICI therapy [14,15]. Badran et al. reported a case of cytomegalovirus pneumonia complicating ICI-induced pneumonitis during pembrolizumab therapy [16]. Sumer et al. described herpes simplex virus pneumonitis in a lung cancer patient treated with immunotherapy (nivolumab/ipilimumab) [17]. Similarly, Foukas et al. documented human herpesvirus 6-related interstitial pneumonitis in a patient with CIP associated with nivolumab [18].

Management of irAEs depends on the severity of toxicity and requires a careful, multidisciplinary approach. Mild (grade 1) events can often be monitored without therapy interruption, while moderate to severe (grade 2–4) toxicities typically require temporary discontinuation of the ICI and initiation of corticosteroids [2,19].

Systemic corticosteroids represent the mainstay of treatment for moderate to severe CIP, as well as for severe CAP with an inflammatory or immune-mediated component. If pneumonitis persists or worsens after 48 h, consider initiating a non-steroidal immunosuppressive agent [2,19].

The decision to resume ICI therapy after resolution of an irAE must be individualized, considering the severity of the initial toxicity, the risk of permanent organ damage, and the presence of contributing factors such as intercurrent infections. Clear criteria for rechallenging patients after severe pneumonitis remain lacking [19].

Recent reports indicate that ICI treatment can be safely resumed in selected patients once the adverse event has resolved, provided that close clinical and laboratory follow-up is ensured [19]. For instance, severe hematologic toxicities, including pure red cell aplasia associated with Parvovirus B19 infection during atezolizumab therapy, were successfully managed with resolution of anemia, and therapy was safely resumed [20]. A previous review showed that rechallenging with ICIs after irAEs was safe in a limited number of cases (15 patients), but no data on durvalumab were reported [21]. In the cohort reported by Lim et al., among 49 patients who developed CIP, 13 were rechallenged with durvalumab, and only one experienced a recurrent low-grade pneumonitis, which led to permanent discontinuation of the drug [9].

In our case, durvalumab was permanently discontinued, and methylprednisolone 40 mg twice daily (2 mg/kg/day) was administered for 10 days, followed by a gradual taper over 4 weeks. Although the typical clinical response to corticosteroids occurs within 48–72 h [2,19], our patient exhibited a slower but steady improvement leading to complete clinical resolution. HMPV infection likely contributed to a more severe clinical course and delayed response to corticosteroid therapy, as evidenced by the prolonged need for high-flow oxygen therapy and the two-week recovery period before significant improvement, suggesting persistent viral-driven inflammation despite adequate immunosuppression.

Finally, during hospitalization, the patient developed leukopenia (nadir WBC 1050/mm^3^), a known hematologic adverse event associated with durvalumab [4]. This condition resolved spontaneously with supportive care and corticosteroid therapy, consistent with an iatrogenic mechanism rather than virus-induced marrow suppression.

However, our case has several limitations. Although HMPV was identified in the lower respiratory tract, its exact pathogenic contribution to lung injury in this case remains uncertain. The detection of viral RNA alone does not confirm causality. However, it is plausible that HMPV acted as a trigger or amplifier of the immune-mediated pneumonitis. Furthermore, the diagnosis of CIP was based on a multidisciplinary clinical–radiologic assessment rather than histopathologic confirmation. Although bronchoalveolar lavage (BAL) can support the suspicion of drug-induced lung injury, it was not performed due to the patient’s clinical improvement following corticosteroid therapy and the difficulty of performing a BAL in the context of high-flow oxygen therapy. Our patient had a history of heavy cigarette smoking but no documented diagnosis of chronic obstructive pulmonary disease or interstitial lung disease, which are considered risk factors for CIP.

Finally, although follow-up imaging at four months showed substantial radiologic improvement, longer-term imaging and functional assessment would be necessary to fully evaluate the reversibility and lasting impact of the lung injury.

## 4. Conclusions

This case underscores the diagnostic complexity and therapeutic challenges of durvalumab-induced pneumonitis in the presence of viral infection. The concomitant detection of HMPV underscores the importance of comprehensive microbiologic testing, including molecular diagnostics, to account for both infectious and immune-mediated lung injury. The prolonged corticosteroid regimen achieved full recovery, demonstrating the effectiveness of timely immunosuppressive therapy even in the setting of concurrent viral infection. Early recognition, prompt steroid initiation, and multidisciplinary management remain crucial to prevent irreversible lung injury and improve patient outcomes.

## Data Availability

Data will be made available upon request.

## Abbreviations

The following abbreviations are used in this manuscript:

BAL	bronchoalveolar lavage
CAP	community-acquired pneumonia
CIP	checkpoint inhibitor pneumonitis
CRP	C-reactive protein
CT	computed tomography
NSCLC	non–small cell lung cancer
HMPV	*human metapneumovirus*
ICI	Immune checkpoint inhibitors
irAEs	immune-related adverse events
PD-1	programmed death 1
PD-L1	programmed death-ligand 1
WBC	White blood cell
